# Anti-NMDAR encephalitis triggered by EBV and HSV-1: A case report and literature review

**DOI:** 10.1097/MD.0000000000043338

**Published:** 2025-07-11

**Authors:** Xiuping Zhan, Xueping Zhang, Xiaoyan Chen, Xiaoyan Niu, Tingting Xuan, Jianhang He, Yazhou Ren, Yue Meng, Tao Guo, Haining Li

**Affiliations:** a Department of Neurology, General Hospital of Ningxia Medical University, Yinchuan, China; b School of the First Clinical Medicine, Ningxia Medical University, Yinchuan, China; c Diagnosis and Treatment Engineering Technology Research Center of Nervous System Diseases of Ningxia Hui Autonomous Region, Yinchuan, China.

**Keywords:** anti-NMDAR encephalitis, autoimmune encephalitis, Epstein-Barr virus, herpes simplex virus, viral encephalitis

## Abstract

**Rationale:**

Anti-N-methyl-d-aspartate receptor (NMDAR) encephalitis is a central nervous system disorder driven by autoantibodies, characterized by a wide range of complex clinical manifestations. Although tumors, such as teratomas, and viral infections have been identified as potential triggering factors, the precise etiology of this condition remains to be elucidated. While herpes simplex virus type 1 (HSV-1) has been linked to the onset of anti-NMDAR encephalitis, cases involving coinfection with Epstein–Barr virus (EBV) and HSV-1 as causative factors are exceedingly rare.

**Patient concerns:**

A 46-year-old female patient who exhibited mental abnormalities, epileptic seizures, and altered consciousness. Analysis of cerebrospinal fluid samples revealed the presence of EBV and HSV-1 infections. The initial autoimmune encephalitis antibody test returned negative results. However, a subsequent test indicated the presence of anti-NMDAR antibodies in the cerebrospinal fluid, whereas serum analysis remained negative.

**Diagnoses:**

The patient was diagnosed with anti-NMDAR encephalitis resulting from infection with the EBV and HSV-1.

**Interventions and Outcomes:**

Antiviral treatments were administered to the patient in conjunction with intravenous methylprednisolone and human immunoglobulin pulse therapy. This initial treatment did not yield satisfactory results, and the patient’s condition remained critical, necessitating the introduction of rituximab for immunosuppressive therapy. Subsequently, the treatment regimen was adjusted to include intravenous tocilizumab, along with an intrathecal injection of 10 mg methotrexate and 10 mg dexamethasone. Ultimately, the patient’s clinical symptoms improved relative to their previous condition, and repeated examinations demonstrated a gradual decrease in the white blood cell count in the cerebrospinal fluid, indicating that the treatment was effective.

**Lessons:**

This case is the first diagnosed at our hospital and represents a rare instance in the literature of anti-NMDAR encephalitis directly triggered by co-infection with EBV and HSV-1. The diagnostic process emphasizes the need to maintain a high level of suspicion and repeat antibody tests in patients suspected of autoimmune encephalitis, even when initial antibody tests are negative.

## 1. Introduction

Anti-*N*-methyl-d-aspartate receptor (NMDAR) encephalitis is an autoimmune disorder of the central nervous system characterized by acute or subacute onset. Clinical manifestations encompass a range of symptoms, including mental and behavioral abnormalities, epileptic seizures, cognitive impairment, movement disorders, and autonomic dysfunction.^[1–5]^ Anti-NMDAR encephalitis was first described by Dalmau et al^[1]^ The etiology of this condition remains unclear; however, research has demonstrated that viral infections are associated with the disorder and may provoke autoimmune responses in the brain.^[6–8]^ Currently, no standardized criteria exist for diagnosing anti-NMDAR encephalitis. In relevant clinical examinations, the detection of NMDAR antibodies in cerebrospinal fluid (CSF) or blood serves as the gold standard for diagnosis.^[9]^ However, brain magnetic resonance imaging (MRI) and electroencephalogram (EEG) findings lack specific characteristics.^[10]^ Viral encephalitis (VE) is an infectious disease of the central nervous system caused by various viruses, with mental and consciousness disorders serving as prominent manifestations.^[11]^ Among these viruses, HSV is often the most frequently implicated.^[11]^ The clinical manifestations and CSF biochemistry of VE and autoimmune encephalitis (AE) patients exhibit significant similarities, which can easily result in misdiagnosis. This report describes the first case of anti-NMDAR encephalitis triggered by Epstein-Barr virus (EBV) and herpes simplex virus type 1 (HSV-1) admitted to our hospital, along with a review of relevant literature to enhance clinicians’ understanding of both VE and anti-NMDAR encephalitis.

## 2. Case presentation

On January 28, 2024, a 46-year-old Chinese woman with no prior history of mental illness presented to our hospital with abnormal behavior, cognitive impairment, altered consciousness, and epileptic seizures. Thirteen days prior to admission, the patient exhibited symptoms such as unexplained crying, nonsensical speech, irritability, and physical aggression. She was treated at a psychiatric facility, where she received oral medication to manage her symptoms; however, there was no improvement. Seven days prior to admission, the patient developed fever and unresponsiveness, which progressed to impaired consciousness, accompanied by facial twitching, a rightward gaze, stiff limbs, and intermittent involuntary shaking of the upper limbs. Upon arrival at our hospital, the physical examination revealed increased muscle tone in the limbs, positive bilateral Babinski signs, and a lack of cooperation during the remainder of the examination. Initial evaluations included a complete blood count that reveals elevated white blood cell (WBC) counts. CSF analysis showed a significant increase in the WBC count, primarily characterized by an increase in lymphocytes. Rheumatological and immunological markers, as well as tumor markers were within normal limits. Subsequently, the patient’s CSF sample was sent to a specialized testing facility in Zhengzhou for high-throughput sequencing of pathogenic microorganism nucleic acids. The test results indicated infections with EBV (sequence number 31, confidence 99%) and HSV-1 (sequence number 3, confidence 70%). Additionally, testing for human immunodeficiency virus, novel coronavirus, influenza A virus, influenza B virus, Treponema pallidum, Mycobacterium tuberculosis, Brucella, Aspergillus, and other pathogens returned negative results. The patient underwent the initial AE antibodies and paraneoplastic antibodies tests on January 30, 2024, both of which were negative. The EEG exhibited moderate abnormalities during dynamic monitoring, with background beta wave changes noted during coma, and frequent partial seizures originating from the left frontal pole and anterior temporal region. Electroneurography revealed abnormal H-reflex responses in both tibial nerves and abnormal F-wave patterns in the bilateral median and tibial nerves. No abnormalities were detected in cranial MRI. Computed tomography scans of the chest and abdomen, along with ultrasound examinations of the reproductive system, showed no evidence of tumors. Based on the patient’s symptoms, clinical signs, and auxiliary examinations, we hypothesized a likely diagnosis of VE. The treatment regimen included ganciclovir for antiviral therapy, and a combination of sodium valproate and levetiracetam for antiepileptic management, along with antipsychotic medications. After initiating treatment, the patient did not experience any further epileptic seizures, and multiple follow-up EEGs revealed no epileptic activity. However, her consciousness was not regained, involuntary movements increased, and autonomic dysfunction was observed. This raised our suspicion for AE, prompting the commencement of intravenous methylprednisolone and human immunoglobulin shock treatment. Concurrently, after thorough communication with the patient’s family, we reevaluated the AE antibodies on February 18, 2024. The test results indicated a positive anti-NMDAR antibody presence in the CSF at a titer of 1:3.2, while serum results remained negative. Consequently, the patient was diagnosed with anti-NMDAR encephalitis. Due to the lack of significant improvement with glucocorticoid and immunoglobulin pulse therapy, and considering the patient’s critical condition, we initiated rituximab immunosuppressive treatment after consulting with our department’s medical team. Unfortunately, the patient’s symptoms did not improve markedly following this intervention. We considered her case to be one of refractory AE with severe infection, leading to a revised treatment plan that included intravenous tocilizumab, as well as intrathecal administration of 10 mg methotrexate and 10 mg dexamethasone. The patient underwent multiple lumbar punctures since admission, with CSF analysis showing a gradual decrease in WBC count (Table 1), and notable improvement in her clinical symptoms. This suggests that our treatment was effective. Regrettably, due to financial constraints, the patient’s family declined further hospitalization in the neurointensive care unit. We will continue to conduct regular follow-ups and monitor her condition closely.

**Table 1:** Intracranial pressure and CSF analysis results of the patient after admission.

Lumbar puncture time	Intracranial pressure (mm H_2_O)	Total cell count (cells/mm³)	WBC count (cells/mm³)	Proportion of lymphocytes (%)	Protein (g/L)	Glucose (mmol/L)	Chloride (mmol/L)	Immunoglobulin IgG (mg/L)
January 30, 2024	125	7360	80	44	0.64	2.0	126	68.60
February 2, 2024	145	90	42	84	0.37	4.0	132	40.50
February 8, 2024	160	60	27	84	0.34	4.0	127	34.20
February 18, 2024	230	40	22	96	0.36	4.0	130	45.20
March 8, 2024	170	80	15	92	0.54	4.0	127	51.00
March 14, 2024	140	30	12	90	0.42	5.0	128	32.80
March 27, 2024	180	24	7	0	0.49	4.0	127	31.80

CSF = cerebrospinal fluid, IgG = immunoglobulin G, WBC = white blood cell.

## 3. Discussion and conclusion

The etiology of anti-NMDAR encephalitis remains unclear; however, studies have indicated a relationship between tumors (particularly teratomas) and viral infections in the pathogenesis of the disease. Various pathogens, including viruses, parasites, bacteria, and spirochetes, have been implicated in the induction of AE, with HSV being the most prevalent.^[6–8]^ EBV and HSV-1 were detected in the CSF of this patient. Anti-NMDAR encephalitis resulting from co-infection with these two viruses is a rare occurrence. Both EBV and HSV-1 are neurotropic viruses, and the precise mechanism by which they induce anti-NMDAR encephalitis remains unclear. We hypothesize that these viruses may lyse neurons, releasing antigens that sensitize antibodies to the NMDAR and thereby trigger an autoimmune response.^[12]^ Approximately 26% to 70% of patients with anti-NMDAR encephalitis exhibit prodromal symptoms or experience prodromal viral infections.^[13]^ The relationship between AE and VE has garnered significant attention. Studies indicate that approximately 27% of patients with herpes simplex encephalitis may develop secondary AE within 2 to 16 weeks following the onset of the virus.^[14]^ Most cases exhibit a bimodal course of encephalitis, while some cases demonstrate an overlapping single-peak pattern.^[14]^ This patient may exhibit a unimodal course of encephalitis, with overlapping stages of anti-NMDAR encephalitis and VE occurring simultaneously. Throughout the disease course, the patient experienced persistent psychoneurological symptoms, without the typical pattern of remission and relapse. However, the WBC count in her CSF gradually decreased (Table 1), and the anti-NMDAR antibodies in the CSF changed from negative to positive, indicating a potential progression of the disease from VE to anti-NMDAR encephalitis. This highlights the challenges associated with the early differentiation between VE and AE.

The gold standard for diagnosing anti-NMDAR encephalitis involves the detection of NMDAR antibodies in either CSF or serum. Clinically, it is important to recognize that AE antibodies in both CSF and blood may be negative during the early stages of the disease, with specific antibodies becoming detectable as the disease progresses. In this case, the patient initially tested negative for AE antibodies; however, a subsequent test revealed positive anti-NMDAR antibodies in the CSF, while serum antibodies remained negative. Previous studies have indicated that the rate of negative serum NMDAR antibody testing among patients with anti-NMDAR encephalitis, despite having NMDAR antibodies in the CSF, is 14%.^[15]^ This finding is supported by the study conducted by Guasp et al, which demonstrated that patients with anti-NMDAR encephalitis who tested positive for CSF antibodies but negative for serum antibodies were older, presented milder neurological symptoms, and had a lower incidence of tumors.^[16]^ Additionally, the diagnosis of anti-NMDAR encephalitis is complicated by the lack of specific characteristics in brain MRI results. The patient in this case underwent 2 MRI examinations during hospitalization, both of which revealed no significant abnormalities. This indicates that, even in patients with severe clinical symptoms of anti-NMDAR encephalitis, MRI examinations may not always identify the underlying lesions. Reports on the frequency of abnormal brain MRI findings in anti-NMDA receptor encephalitis vary widely, ranging from 11% to 83%.^[17]^ The underlying reasons for this variability remain unclear and warrant further investigation.

In summary, EBV and HSV-1 can lead to anti-NMDAR encephalitis. The clinical manifestations and CSF biochemistry of VE and AE patients are often very similar, which may result in missed or incorrect diagnoses. Most cases of AE following VE exhibit a clinical phenotype characterized by bimodal encephalitis, while a minority present with a unimodal phenotype. Clinicians should remain vigilant for patients whose CSF and blood NMDAR antibodies are initially negative, as these may become positive during the disease course. If the disease is highly suspected, repeated AE antibody testing is warranted. Patients with anti-NMDAR encephalitis may show negative results in serum anti-NMDAR antibody tests despite the presence of these antibodies in CSF. The reasons for inconsistent antibody test results between CSF and serum remain unclear. Anti-NMDAR encephalitis typically features an acute onset and rapid progression, complicating diagnosis. As clinicians, we must enhance our understanding of this disease to ensure early detection, accurate diagnosis, and timely treatment to improve patient prognosis.

## Acknowledgments

We would like to express our sincere gratitude to our patient and her family for their cooperation in the preparation of this report.

## Author contributions

**Conceptualization:** Tao Guo, Haining Li.

**Data curation:** Xiuping Zhan, Xueping Zhang.

**Project administration:** Xiaoyan Chen, Xiaoyan Niu, Tingting Xuan, Jianhang He, Yazhou Ren, Yue Meng.

**Resources:** Xueping Zhang.

**Supervision:** Tao Guo, Haining Li.

**Writing – original draft:** Xiuping Zhan.

**Writing – review & editing:** Xiaoyan Chen, Xiaoyan Niu.
